# Supplementary material for: Essential Oils: A Natural Weapon against Antibiotic-Resistant Bacteria Responsible for Nosocomial Infections
Source: Antibiotics (Basel). 2021 Apr 10;10(4):417. doi: 10.3390/antibiotics10040417 (PMC8070240; doi:10.3390/antibiotics10040417)
Supplement: Supplementary file 1 [file antibiotics-10-00417-s001.pdf]

Table S1. Synergistic activity resulted mixing EO/EO and Antibiotic/EO in VRE strains, by FIC Index calculation. MIC values are expressed in μg/mL.

**Strains**	**Drug /EO**	**MIC alone**	**MIC combined**	**FICI**	**Strains**	**Drug /EO**	**MIC alone**	**MIC combined**	**FICI**
**A29**	EEO	8	2	0.26	**B5**	TTO	8	0.25	0.03
	VAN	256	2			VAN	256	0.5	
	TTO	1	0.25	0.25		CEO	64	16	0.5
	VAN	256	1			VAN	256	64	
	AEO	256	64	0.5	**VAN 3**	EEO	16	0.25	0.18
	VAN	256	64			VAN	256	0.5	
	LEO	64	16	0.5		EEO	16	2	0.13
	VAN	256	64			VAN	256	0.25	
	CEO	32	8	0.5		TTO	64	16	0.5
	VAN	256	64			VAN	256	64	
	AEO	256	64	0.5		CEO	64	16	0.5
	CEO	32	8			VAN	256	64	
	EEO	8	2	0.5		EEO	16	4	0.5
	TTO	1	0.25			TTO	64	16	
**A30**	EEO	8	0.25	0.03	**VAN 4**	CEO	128	32	0.5
	VAN	256	1			VAN	256	64	
	TT0	16	0.25	0.02		TTO	8	2	0.5
	VAN	256	2			CEO	128	32	
	CEO	128	32	0.5	**VAN 5**	TTO	32	8	0.5
	VAN	256	64			VAN	16	4	
					**VAN 19**	CEO	256	64	0.5
						VAN	8	2	

Table S2. Synergistic activity resulted mixing EO/EO and Antibiotic/EO in MRSA strains, by FIC Index calculation. MIC values are expressed in μg/mL.

**Strains**	**Drug /EO**	**MIC alone**	**MIC combined**	**FICI**	**Strains**	**Drug /EO**	**MIC alone**	**MIC combined**	**FICI**
**12A**	LEO	32	8	0.5	**MRSA**	TTO	8	0.25	0.04
	OXA	256	64			OXA	256	1	
	EEO	64	16	0.5		EEO	32	0.5	0.18
	OXA	256	64			OXA	256	0.5	
	TTO	32	8	0.5		AEO	128	32	0.5
	OXA	256	64			OXA	256	64	
	EEO	64	16	0.5		AEO	128	32	0.5
	TTO	32	8			EEO	32	8	
**12 B**	LEO	256	64	0.5		AEO	128	32	0.5
	OXA	256	64			TTO	8	2	
**C1**	EEO	8	0.25	0.04		EEO	32	8	0.5
	OXA	256	2			TTO	8	2	
	EEO	8	2	0.5	**MRSA 1**	TTO	32	8	0.5
	TTO	4	1			OXA	4	1	
**C3**	TTO	8	0.25	0.03		TTO	32	8	0.5
	OXA	256	0.25			CEO	512	128	
	TTO	8	1	0.13	**MRSA 2**	TTO	16	2	0.375
	OXA	256	0.5			OXA	4	1	
	EEO	32	8	0.5		EEO	8	2	0.5
	OXA	256	64			TTO	16	4	
	EEO	32	8	0.5	**O**	TTO	8	0.25	0.03
	TTO	8	2			OXA	256	0.5	
						EEO	32	8	0.375
						TTO	8	1	

Table S3. Synergistic activity resulted mixing EO/EO and Antibiotic/EO in ESBL-producing *E. coli* strains, by FIC Index calculation. MIC values are expressed in μg/mL.

Strains	Drug /EO	MIC alone	MIC combined	FICI	Strains	Drug /EO	MIC alone	MIC combined	FICI
**34**	TTO	8	0.25	0.04	**22CT**	LEO	256	64	0.5
	CTX	32	0.25			CTX	64	16	
	LEO	256	64	0.5		CEO	64	16	0.5
	CTX	32	8			CTX	64	16	
	CEO	64	16	0.5		LEO	256	64	0.5
	CTX	32	8			CEO	64	16	
	LEO	256	64	0.5		TTO	4	1	0.5
	TTO	8	2			CEO	64	16	
	TTO	8	2	0.5	**23CT**	TTO	2	0.25	0.18
	CEO	64	16			CTX	64	4	
**39**	CEO	32	8	0.5	**40CT**	TTO	4	0.25	0.07
	CTX	64	16			CTX	32	0.25	
**22A**	TTO	8	2	0.27		LEO	256	64	0.5
	CTX	64	1			CTX	32	8	
	LEO	512	128	0.5	**23DT**	TTO	2	0.5	0.27
	CTX	64	16			CTX	64	1	
	CEO	32	8	0.5		CEO	512	128	0.5
	CTX	64	16			CTX	64	16	
	LEO	512	128	0.5		TTO	2	0.5	0.5
	TTO	8	2			CEO	512	128	
	TTO	8	2	0.5	**31DT**	TTO	1	0.25	0.26
	CEO	32	8			CTX	32	0.25	
**26A**	TTO	1	0.25	0.26		LEO	256	64	0.5
	CTX	32	0.25			CTX	32	8	
**28A**	TTO	4	0.25	0.07		CEO	64	16	0.5
	CTX	32	0.25			CTX	32	8	
	LEO	256	64	0.5		LEO	256	64	0.5
	CTX	32	8			CEO	64	16	
	EEO	64	16	0.5	**45DT**	EEO	128	32	0.5
	CTX	32	8			CTX	32	8	
	CEO	64	16	0.5		CEO	128	32	0.5
	CTX	32	8			CTX	32	8	
	LEO	256	64	0.5		EEO	128	32	0.5
	CEO	64	16			TTO	1	0.25	
	EEO	64	16	0.5	**22F**	TTO	2	0.25	0.13
	TTO	4	1			CTX	64	0.25	
	TTO	4	1	0.5		LEO	512	128	0.5
	CEO	64	16			CTX	64	16	
**38A**	TTO	4	0.25	0.07		CEO	64	16	0.5
	CTX	32	0.25			CTX	64	16	
	LEO	256	64	0.5		LIM	512	128	0.5
	CTX	32	8			CEO	64	16	
	CEO	128	32	0.5		TTO	2	0.5	0.5
	CTX	32	8			CEO	64	16	
	TTO	4	1	0.5	**23F**	TTO	1	0.25	0.25
	CEO	128	32			CTX	64	0.25	
**41A**	TTO	4	0.25	0.07		LEO	128	32	0.5
	CTX	32	0.25			CTX	64	16	
	LEO	256	64	0.5		LEO	128	32	0.5
	CTX	32	8			TTO	1	0.25	
**36AT**	LEO	512	128	0.5	**31FT**	LEO	512	128	0.5

	CTX	32	8	
	LEO	512	128	0.5
	TTO	2	0.5	
**22BT**	LEO	256	64	0.5
	CTX	64	16	
	EEO	32	8	0.5
	CTX	64	16	
	TTO	128	32	0.5
	CTX	64	16	
	CEO	128	32	0.5
	CTX	64	16	
	LEO	256	64	0.5
	EEO	32	8	
	LEO	256	64	0.5
	TTO	128	32	
	LEO	256	64	0.5
	CEO	128	32	
**36BT**	TTO	1	0.25	0.25
	CTX	64	0.25	
**24C**	TTO	256	64	0.5
	CTX	8	2	
	CTX	64	16	
	EEO	256	64	0.5
	CTX	64	16	
	EEO	256	64	0.5
	TTO	16	4	
**27G**	TTO	2	0.25	0.14
	CTX	64	1	
	LEO	512	128	0.5
	CTX	64	16	
	CEO	64	16	0.5
	CTX	64	16	
**9 Cl**	TTO	4	1	0.27
	CTX	16	0.25	
	LEO	512	128	0.5
	CTX	16	4	
	CEO	512	128	0.5
	CTX	16	4	
	LEO	512	128	0.5
	TTO	4	1	
	TTO	4	1	0.5
	CEO	512	128	
**11 Cl**	AEO	512	128	0.5
	CTX	32	8	
